# Glucose Homeostasis Variables in Pregnancy *versus* Maternal and Infant Body Composition

**DOI:** 10.3390/nu7075243

**Published:** 2015-07-10

**Authors:** Pontus Henriksson, Marie Löf, Elisabet Forsum

**Affiliations:** 1 Department of Clinical and Experimental Medicine, Linköping University, Linköping SE 581 85, Sweden; E-Mail: pontus.henriksson@liu.se; 2 Department of Biosciences and Nutrition, Karolinska Institute, NOVUM, Huddinge SE 141 83, Sweden; E-Mail: marie.lof@ki.se

**Keywords:** body composition, infant, insulin resistance, pregnancy, sex difference

## Abstract

Intrauterine factors influence infant size and body composition but the mechanisms involved are to a large extent unknown. We studied relationships between the body composition of pregnant women and variables related to their glucose homeostasis, *i.e.*, glucose, HOMA-IR (homeostasis model assessment-insulin resistance), hemoglobin A_1c_ and IGFBP-1 (insulin-like growth factor binding protein-1), and related these variables to the body composition of their infants. Body composition of 209 women in gestational week 32 and of their healthy, singleton and full-term one-week-old infants was measured using air displacement plethysmography. Glucose homeostasis variables were assessed in gestational week 32. HOMA-IR was positively related to fat mass index and fat mass (*r*^2^ = 0.32, *p* < 0.001) of the women. Maternal glucose and HOMA-IR values were positively (*p* ≤ 0.006) associated, while IGFBP-1was negatively (*p* = 0.001) associated, with infant fat mass. HOMA-IR was positively associated with fat mass of daughters (*p* < 0.001), but not of sons (*p* = 0.65) (Sex-interaction: *p* = 0.042). In conclusion, glucose homeostasis variables of pregnant women are related to their own body composition and to that of their infants. The results suggest that a previously identified relationship between fat mass of mothers and daughters is mediated by maternal insulin resistance.

## 1. Introduction

Overweight and obesity in childhood are severe health problems [1] and factors early in life, for example a high birth weight [2], may promote their development. Back in 1952, Pedersen [3] proposed that the high blood glucose concentration in gestational diabetes mellitus (GDM) is transmitted to the fetus with a subsequent stimulation of fetal growth and fat retention. The HAPO-study [3] confirmed that hyperglycemia, less severe than in GDM, is also associated with such effects in the offspring. Recently, we found evidence for a relationship between body fatness of pregnant women and body fatness of their infant daughters, while no such relationship was found for sons [4]. Lingwood *et al.* [5] identified a similar difference between boys and girls born to women with GDM. The mechanism behind such a sex difference is unknown, but it may be found in factors of relevance in relation to associations between maternal fatness and infant size or body composition. Previous studies have shown relationships between estimates of body fatness of pregnant women and concentrations of glucose and insulin [6], as well as of the insulin-like growth factor binding protein 1 (IGFBP-1) [7], in their circulation. As mentioned above, published research indicates that glucose homeostasis during pregnancy influences fetal growth and infant body composition [3], and the concentration of IGFBP-1 in the circulation of pregnant women has been linked to fetal growth [8,9] but there are no studies regarding its relationship with infant body composition. This protein is of interest since its concentration in the circulation is inversely correlated with that of insulin and it is involved in glucose homeostasis [10]. Studies in this area are often based on body mass index, a relatively poor estimate of the proportion of fat in the body, especially during pregnancy [11]. In our previous study [4] body composition of pregnant women and infants was assessed by means of air displacement plethysmography, a more accurate estimate of body fatness.

Studies relating glucose homeostasis variables in pregnant women to infant size and body fatness tend to show that such relationships are relatively weak explaining, for example, only about 5% of the variation in birth weight [12]. Nevertheless, such variables may be relevant in a population perspective. Consequently, variables such as glucose, homeostasis model assessment-insulin resistance (HOMA-IR), IGFBP-1 and hemoglobin A_1c_ (HbA_1c_) in the maternal circulation are of potential interest in relation to offspring size and body composition. The first aim of this paper was to study the body composition of healthy pregnant women *versus* glucose, HOMA-IR, HbA_1c_ and IGFBP-1 in their circulation. A second aim was to study these variables in relation to size and body composition of infants born to these women, and our third aim was to identify mechanisms mediating the previously reported relationship [4] between mothers and daughters regarding body fatness.

## 2. Experimental Section

### 2.1. Participants and Study Outline

Pregnant women were recruited between 2008 and 2010 from a well-educated middle income Swedish population as previously described [4]. At gestational week 32, their body composition was assessed and blood samples were collected after an overnight fast. Gestational age was assessed based on an ultrasound examination in gestational week 12–14 [13]. When the woman had delivered, the body composition of her infant was assessed at one week of age. Healthy, singleton and full-term (≥37 gestational weeks at birth) infants born to mothers without preeclampsia were included. The mothers were considered to be non-diabetic during pregnancy and none of them received treatment for GDM. Preeclampsia was defined as a blood pressure ≥ 140/90 mm Hg, or an increase during pregnancy in diastolic blood pressure ≥ 25 mm Hg combined with proteinuria. The study included 209 mother-infant units with blood samples from the mothers and body composition data for the women and infants. Detailed information about the subjects in this cohort is found elsewhere [4]. The study was approved by the Ethics Committee in Linköping on 12 December 2007 (187–07).

### 2.2. Body Composition of Women in Gestational Week 32

Height was measured with a wall stadiometer to the nearest 0.5 cm. The Bod Pod (COSMED USA, Inc., Concord, CA, USA) was used to assess body weight and body volume (by means of air displacement plethysmography) as previously described [14]. Thoracic gas volume was predicted using the Bod Pod software 4.2.4, which yields satisfactory body composition results in gestational week 32 [14]. Body composition was calculated using the two-component model and the fat-free mass density value by van Raaij *et al.* [15] for women in gestational week 32. This value has been confirmed in a group of Swedish women in gestational week 32 who were similar to those in the present study regarding age and body mass index [16]. As previously discussed [4], the validity of the two-component model in gestational week 32 is thus well documented. Fat mass index was (fat mass (kg)/height^2^ (m)) and fat-free mass index was (fat-free mass (kg)/height^2^ (m)).

### 2.3. Glucose Homeostasis Variables of Women in Gestational Week 32

Blood was collected in EDTA-containing vacutainer tubes for high-performance liquid chromatography analysis of HbA_1c_ [17]. Plasma *glucose* was analyzed by means of the glucose hexokinase method and serum insulin was analyzed using the Elecsys electrochemiluminescence immunoassay on a Cobas 602 (Roche Diagnostics Scandinavia AB, Bromma, Sweden). HOMA-IR was calculated according to Matthews *et al.* [18]. Serum samples were stored at −70 °C prior to analysis of IGFBP-1 using a one-step enzyme-linked immunosorbent assay (R & D Systems, Minneapolis, MN, USA). Inter assay coefficients of variation were 20.0% and 7.8% for low (4 ng/mL) and high (1688 ng/mL) IGFBP-1 concentrations, respectively. Glucose, insulin and HbA_1c_ were analyzed at the Department of Clinical Chemistry, Linköping University Hospital, which is accredited for these analyses (ISO/IEC 17025).

### 2.4. Body Size and Composition of Infants

Infant length was measured to the nearest 0.5 cm. Subsequently, infants were weighed without clothing and their body volume was measured using air displacement plethsmography (Pea Pod, COSMED USA, Inc., Concord, CA, USA) and body composition was calculated as previously described [4]. This technique is safe, rapid and, although not a reference method, its validity in infants is well documented [19].

### 2.5. Statistics

Glucose homeostasis variables were log transformed to obtain normality since they were positively skewed. Internal standard deviation scores (SDS) were created by subtracting the sample mean from each observation and dividing the difference by the standard deviation (SD) of the sample. Relationships between body composition (independent) variables and glucose homeostasis (dependent) variables for women in gestational week 32 were analyzed using simple regression [20]. Correlations were compared as described by Kleinbaum *et al.* [20]. Relationships between glucose homeostasis (independent) variables and infant size and body composition (dependent) variables were analyzed by means of multiple regression [20]. Each glucose homeostasis variable was fitted in a separate model, adjusted for infant sex, maternal parity, infant gestational age at birth and age at measurement. To identify sex differences in relationships between glucose homeostasis variables and infant fat mass, interaction terms were created [20] by multiplying each glucose homeostasis variable by infant sex. Interaction terms were entered as separate independent variables in regression models and when significant, separate models (adjusted for maternal parity, infant gestational age at birth and age at measurement) were created for boys and for girls. Adjusting regression models for fewer or additional variables (mode of feeding, maternal age and education level) had very little effect on the results. Results were also very similar if fat mass (%) rather than fat mass (g) of infants was used. Results based on insulin were very similar to those presented for HOMA-IR in the paper and therefore such results are not shown. All regression models were examined to confirm that required assumptions for regression [20] were not violated. Regression (*b*) and correlation (*r*) coefficients were calculated. For multiple regression models, *r* represents the partial correlation coefficient. *p* < 0.05 was statistically significant. All hypothesis tests were two-sided. Statistical analysis was performed using PASW Statistics 18 (IBM, Sowers, NY, USA) or Statistica software 9.1 (StatSoft Inc., Tulsa, OK, USA). Values given are means and SDS.

## 3. Results

### 3.1. Characteristics of Mothers and Their Infants

Information about the women and infants is given in Table 1. When values regarding glucose, insulin, HOMA-IR, HbA_1c_ and IGFBP-1 for mothers of girls are compared to the corresponding values for mothers of boys, no significant differences are found. The fasting plasma glucose concentration was ≥5.1 mmol/L in 46 (22%) women.

### 3.2. Glucose Homeostasis Variables vs. Body Composition of Women in Gestational Week 32

Table 2 shows results obtained when glucose homeostasis variables (glucose, HOMA-IR, HbA_1c_, and IGFBP-1) of women in the study were regressed on their fat mass, fat mass index and fat-free mass index. Each glucose homeostasis variable was significantly related to all these body composition variables. As shown in the table, the coefficients of determination (*r*^2^) for fat-free mass index and fat mass index were similar for glucose, HbA_1c_ and IGFBP-1. Relationships between variables describing body fatness (fat mass and fat mass index) *vs.* HOMA-IR were particularly strong (*r*^2^ = 0.32), and their correlation coefficients were significantly higher than the corresponding value for fat-free mass index (*r*^2^ = 0.14) (Table 2). When variables describing body composition were correlated with HOMA-IR results similar to those obtained for all women (Table 2) were found for women with only male or only female fetuses. When IGFBP-1 (SDS) was regressed on insulin (SDS) we observed a significant negative relationship (*b* = −0.55, *r*^2^ = 0.30, *n* = 204, *p* < 0.001).

**Table 1:** Characteristics of women and infants at the times of measurement.

Women	All ( *n* = 209)	Mothers of Girls	Mothers of Boys
		( *n* = 99)	( *n* = 110)
Stage of Gestation (weeks)	31.4 ± 0.3	31.4 ± 0.3	31.4 ± 0.3
Body mass index (kg/m^2^)	26.6 ± 3.4	26.5 ± 3.7	26.7 ± 3.0
Fat-free mass index (kg/m^2^)	17.3 ± 1.4	17.4 ± 1.5	17.3 ± 1.4
Fat mass index (kg/m^2^)	9.3 ± 2.6	9.2 ± 2.8	9.4 ± 2.4
Fat-free mass (kg)	49.6 ± 5.3	49.8 ± 5.5	49.4 ± 5.1
Fat mass (kg)	26.5 ± 7.4	26.2 ± 7.8	26.7 ± 7.1
Fat mass (%)	34.3 ± 5.8	33.9 ± 6.2	34.6 ± 5.5
Glucose in plasma (mmol/L)	4.8 ± 0.4	4.9 ± 0.4	4.8 ± 0.3
Insulin in serum (pmol/L)	66 ± 37	65 ± 38	67 ± 36
HOMA-IR	2.1 ± 1.3	2.1 ± 1.4	2.1 ± 1.2
HbA_1c_ * (mmol/mol)	31 ± 3	31 ± 3	32 ± 3
IGFBP-1 in serum ^†^ (ng/mL)	108 ± 71	110 ± 81	107 ± 62
**Infants**	**All (*n* = 209)**	**Girls (*n* = 99)**	**Boys (*n* = 110)**
Age (weeks)	1.0 ± 0.3	1.0 ± 0.3	1.1 ± 0.2
Length (cm)	51.5 ± 1.5	51.0 ± 1.5	52.0 ± 1.5
Weight (g)	3591 ± 458	3523 ± 445	3653 ± 462
Fat-free mass (g)	3151 ± 343	3049 ± 318	3243 ± 339
Fat mass (g)	440 ± 183	474 ± 171	409 ± 188
Fat mass (%)	12.0 ± 4.0	13.2 ± 3.6	10.9 ± 4.1

Data are means ± SD. HOMA-IR, homeostatic model assessment-insulin resistance; HbA_1c_ hemoglobin A_1c_; IGFBP-1, insulin-like growth factor binding protein 1. * *n* = 208 (98 mothers of girls and 110 mothers of boys); ^†^ *n* = 204 (97 mothers of girls and 107 mothers of boys).

### 3.3. Glucose Homeostasis Variables of Women in Gestational Week 32 vs. Size and Body Composition of Their Infants

**Figure 1:**
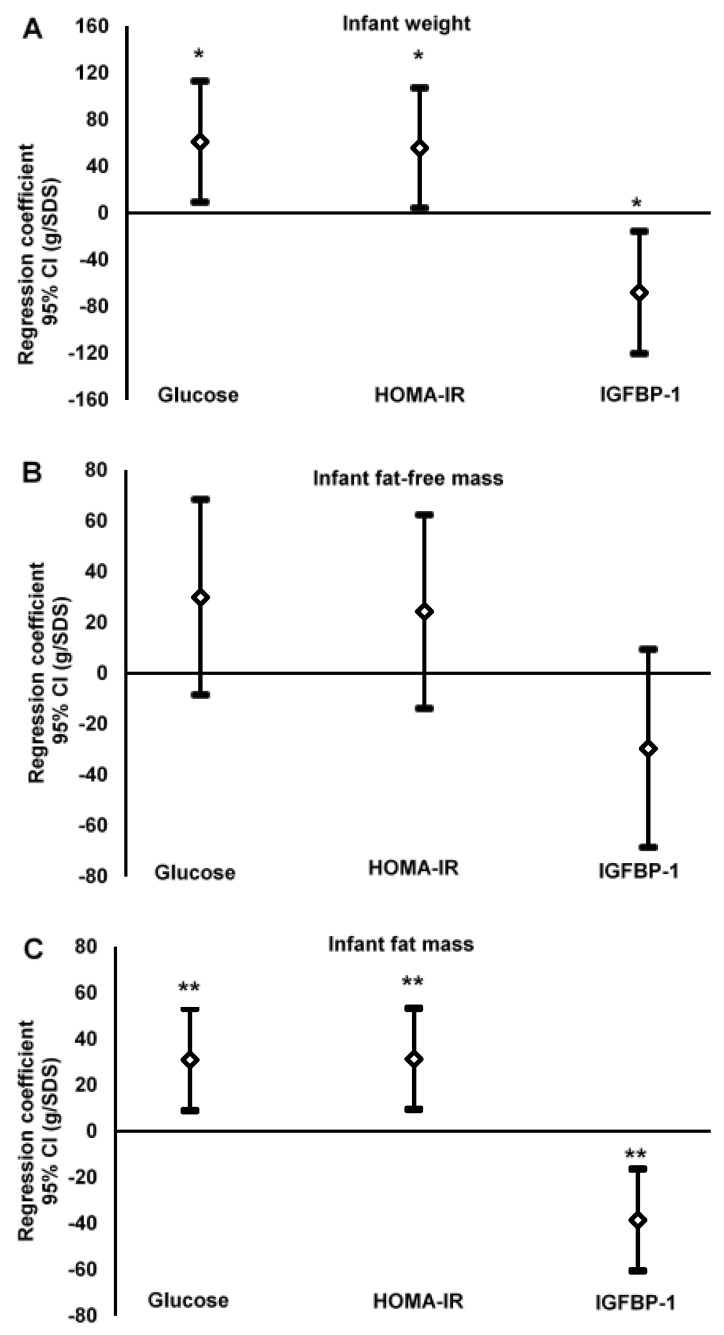
Weight (g), fat-free mass (g) and fat mass (g) of infants at one week of age (dependent variables) regressed on glucose homeostasis variables (glucose, HOMA-IR and IGFBP-1), assessed in the circulation of their mothers when pregnant in gestational week 32 (independent variables). Glucose homeostasis variables are expressed as standard deviation scores (SDS) and estimates are presented as regression coefficients (*b*) in g/SDS with a 95% confidence interval (CI). Regression models were adjusted for maternal parity, infant gestational age at birth, infant sex, and age at measurement. HOMA-IR (homeostasis model assessment-insulin resistance); IGFBP-1 (insulin-like growth factor binding protein-1). Differences between regression coefficient and zero: * *p* < 0.05, ** *p* < 0.01, *** *p* < 0.001.

Infant length was not related (*p* ≥ 0.18) to any of the investigated glucose homeostasis variables, and no significant relationships (*p* ≥ 0.89) were observed between HbA_1c_ and any variable describing infant size or body composition. Figure 1 shows relationships between maternal glucose, HOMA-IR and IGFBP-1 on the one hand, and infant weight (A), infant fat-free mass (B) and infant fat mass (C) on the other hand. Regression coefficients for relationships with infant weight *versus* glucose and HOMA-IR, respectively, were positive and significant. The corresponding relationship between infant weight and IGFBP-1 was also significant but negative. In contrast, none of the glucose homeostasis variables studied was significantly related to infant fat-free mass. However, the regression coefficients for the relationships with infant fat mass *versus* glucose and HOMA-IR, respectively, were positive and significant, while the corresponding relationship with IGFBP-1 was significant and negative.

**Table 2:** Glucose homeostasis variables (glucose, HOMA-IR, HbA_1c_ and IGFBP-1) in the circulation of women, pregnant in gestational week 32, regressed on variables describing their body composition (fat mass, fat mass index and fat-free mass index). (Slope of regression line (*b*) and coefficient of determination (*r*^2^), calculated from *r* *).

Body Composition ^†^	Glucose (SDS ^‡^)			HOMA-IR (SDS ^‡^)			HbA_1c_ (SDS ^‡^)			IGFBP-1 (SDS ^‡^)		
(Independent Variables)	*n* = 209			*n* = 209			*n* = 208			*n* = 204		
	*b*	*r* ^2^	*p*	*b*	*r* ^2^	*p*	b	*r* ^2^	*p*	*b*	*r* ^2^	*p*
Fat mass (kg)	0.04	0.07	<0.001	0.08	0.32 ^§^	<0.001	0.02	0.03	0.012	−0.05	0.14	<0.001
Fat mass index (kg/m^2^)	0.10	0.07	<0.001	0.22	0.32 ^§^	<0.001	0.08	0.04	0.003	−0.14	0.14	<0.001
Fat-free mass index (kg/m^2^)	0.22	0.10	<0.001	0.26	0.14 ^§^	<0.001	0.16	0.05	0.001	−0.26	0.13	<0.001

HOMA-IR, homeostatic model assessment-insulin resistance; HbA_1c_ hemoglobin A_1c_, IGFBP-1, insulin-like growth factor binding protein 1; SDS, standard deviation score; * Analyzed using simple regression analysis; ^†^ Body composition of pregnant women was measured in gestational week 32; ^‡^ Internal SDS, calculated as described in the experimental section; ^§^ Correlation coefficients between variables describing body fatness (*i.e.*, fat mass and fat mass index) on the one hand, and HOMA-IR on the other hand, were significantly (*p* ≤ 0.005) higher than the corresponding value for fat-free mass index *vs.* HOMA-IR.

### 3.4. HOMA-IR of Women in Gestational Week 32 vs. Fat Mass of Their Infant Sons and Daughters

**Figure 2:**
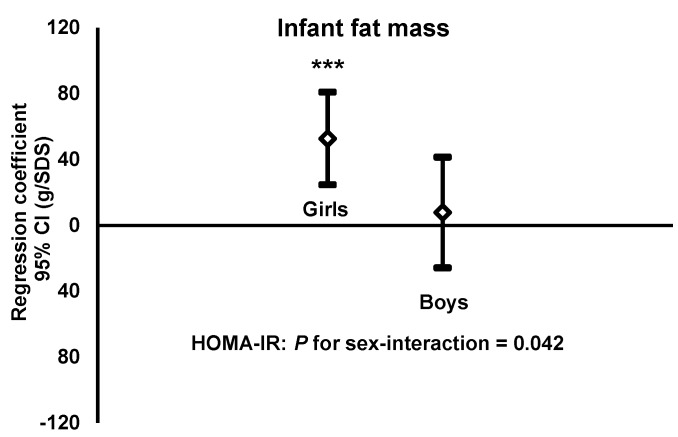
Fat mass (g) of one-week-old girls and boys (dependent variables) regressed on HOMA-IR, expressed as standard deviation score (SDS), of their mothers when pregnant in gestational week 32 (independent variables). Estimates are presented as regression coefficients (*b*) in g/SDS with a 95% confidence interval (CI). Regression models were adjusted for maternal parity, infant gestational age at birth and age at measurement. HOMA-IR (homeostasis model assessment-insulin resistance). Differences between regression coefficient and zero: * *p* < 0.05, ** *p* < 0.01, *** *p* < 0.001. Interaction term (HOMA-IR × infant sex) obtained as described in the experimental section.

When infant fat mass was the dependent variable, we observed a significant sex interaction for maternal HOMA-IR (*p* = 0.042), but not for maternal glucose (*p* = 0.33) or IGFBP-1 (*p* = 0.93). Consequently, we investigated relationships between HOMA-IR and infant fat mass for girls and boys separately. These relationships were positive and significant for girls but not significant for boys (Figure 2).

### 3.5. Fat Mass of Infant Girls vs. Fat Mass and HOMA-IR of Their Mothers in Gestational Week 32

Table 3 shows an analysis in which fat mass of infant girls is regressed on the fat mass and HOMA-IR of their pregnant mothers. Maternal fat mass and HOMA-IR are both positively and significantly related to the fat mass of their daughters (models A and B). However, when including both maternal fat mass and HOMA-IR as independent variables in a regression analysis (model C), only the relationship with HOMA-IR remains significant. The adjusted *r*^2^ is similar for all models in Table 3. These results suggest that the insulin resistance associated with body fatness during pregnancy represents a mechanism that is mediating the previously identified [4] relationship between mothers and daughters regarding fat mass.

**Table 3:** Fat mass of one-week-old girls regressed on fat mass and HOMA-IR of their mothers when pregnant in gestational week 32. (Slope of regression line (*b*) and coefficient of determination (*r*^2^), calculated from r *).

Model	Maternal (Independent) Variables	*b*	*r* ^2^	*p*	Model Adjusted *r*^2^
A *	Fat mass (kg)	5.8	0.09	0.004	0.27
B *	HOMA-IR (SDS ^†^)	52.7	0.13	<0.001	0.30
C *	Fat mass (kg)	2.7	0.01	0.24	0.31
	HOMA-IR (SDS ^†^)	41.4	0.06	0.017	

SDS, standard deviation score; HOMA-IR, homeostatic model assessment-insulin resistance; * Analyzed using multiple regression analysis with fat mass (g) of girls as the dependent variable; Models also included maternal parity, infant gestational age at birth and age at measurement as independent variables; ^†^ Internal SDS, calculated as described in the experimental section.

(**A**) Infant weight
  glucose: *b* = 61.0, *r*^2^ = 0.03, *n* = 209, *p* = 0.022
  HOMA-IR: *b* = 55.6, *r*^2^ = 0.02, *n* = 209, *p* = 0.035
  IGFBP-1: *b* = −68.2, *r*^2^ = 0.03, *n* = 204, *p* = 0.011
(**B**) Infant fat-free mass
  glucose: *n* = 209, *p* = 0.13
  HOMA-IR: *n* = 209, *p* = 0.21
  IGFBP-1: *n* = 204, *p* = 0.13
(**C**) Infant fat mass
  glucose: *b* = 31.0, *r*^2^ = 0.04, *n* = 209, *p* = 0.006
  HOMA-IR: *b* = 31.3, *r*^2^ = 0.04, *n* = 209, *p* = 0.005
  IGFBP-1: *b* = −38.5, *r*^2^ = 0.06, *n* = 204, *p* = 0.001

HOMA-IR
  Girls: *b* = 52.7, *r*^2^ = 0.13, *n* = 99, *p* < 0.001
  Boys: *b* = 7.8, *r*^2^ = 0.00, *n* = 110, *p* = 0.65.

## 4. Discussion and Conclusions

As previously discussed [4], the weight and length of the infants in this study were similar to corresponding figures for healthy Swedish infants, while the proportions of overweight and obesity of their mothers were slightly lower than such figures for Swedish women in general. Although our women were considered to be non-diabetic, 22% had a fasting plasma glucose concentration equal to or above 5.1 mmol/L, a concentration suggested to indicate GDM if present in gestational weeks 24–28 [21]. Our women were investigated later in gestation, but they undoubtedly had high plasma glucose concentrations. This is obvious when comparing their fasting glucose concentrations to corresponding figures in other populations [3,6,12]. Their values for insulin were slightly higher than those published by Friis *et al.* [6], while the concentration of IGFBP-1 in serum was slightly lower than previously reported values [8,9].

All glucose homeostasis variables studied were correlated not only with the fat mass index of the women but also with their fat-free mass index. However, for HOMA-IR, the correlation with the fat mass index was stronger than that with the fat-free mass index. This is in agreement with the contention that an effect of body fatness (as in obesity) is to increase insulin resistance and the concentration of insulin in blood. We have previously identified relationships between body fatness and HOMA-IR in women and suggested that this relationship is enhanced in gestational week 32 [22].

Our finding regarding a possible mechanistic role of insulin resistance influencing the body composition of baby girls is of interest in relation to the hypothesis presented by Watve and Yajnik [23] suggesting that insulin resistance is a key factor regulating life history strategies relevant for reproductive outcome and later health. Our results certainly represent the kind of data needed to develop this hypothesis. If verified, it may have the potential to revolutionize prevention and treatment of disorders associated with insulin resistance possibly with important consequences for public health [23].

We have previously reported a relationship between body fatness of mothers and newborn daughters while no such relationship was found for newborn sons [4]. In the present study, conducted in the same cohort, we found evidence suggesting that this observation is explained by a difference between boys and girls with respect to how they respond to maternal insulin resistance. In this context, it may be relevant to note a report [24] showing a significant relationship between the plasma glucose concentration in pregnancy and placental weight in women carrying a female fetus but not in women with a male fetus. The weight of the placenta is associated with its capacity to transport glucose and other nutrients from mother to fetus [25]. A larger placenta will thus tend to increase glucose, and subsequently insulin, in the fetal circulation. Consequently, the effect of a high plasma glucose concentration in the mother will be more pronounced in the female than in the male fetus. Furthermore, Simón-Muela *et al.* [26] reported that insulin concentrations in cord blood were positively related to skinfold thickness in newborn girls, but not in newborn boys. These proposed mechanisms are certainly speculative and need further studies. Such studies should include measurements of placental weight and relevant components in cord blood.

IGFBP-1 is part of the insulin-like growth factor- (IGF) system, a mediator of somatic growth, and is one of six binding proteins forming complexes with IGF-I and IGF-II [10]. Our study confirmed the inverse relationship between blood concentrations of insulin and IGFBP-1 and previous reports [8,9] showing that the concentration of IGFBP-1 in the maternal circulation is inversely related to the birth weight of the infant. We found that high such concentrations were associated with less fat-free mass and less fat mass of the infant, although only the association with fat mass was significant. These results may be explained by a specific effect of IGFBP-1 on fat retention by the fetus and/or by a general effect on fetal growth. We suggested [8] that a finely tuned balance among components of the IGF system provides a means for fetal growth regulation in response to the maternal nutritional status. The specific mechanisms behind such a regulation are, however, unknown.

A strength of this study is that, to our knowledge, it is the first to present accurate estimates of maternal and infant body composition in combination with data describing glucose homeostasis during pregnancy. In addition, the study was large enough to identify a significant sex interaction regarding the relationship between HOMA-IR of pregnant women and infant fat mass. This is a more satisfactory way to identify a sex difference than only presenting data for boys and girls separately. Finally, as pointed out before [4], measurements were performed within a narrow time frame, especially important in early infancy when size and body composition change rapidly.

A possible limitation is that the fetus is part of a woman’s body in gestational week 32. However, the contributions of fat and fat-free mass by the fetus to the body composition of a woman at this stage of gestation are small [4]. Therefore, it is unlikely that these contributions have affected our conclusions [4]. Furthermore, we have used HOMA-IR to estimate insulin resistance. Although widely used HOMA-IR is only a surrogate measure and thus our findings require confirmation using a more accurate assessment of insulin resistance. Due to the observational design of this study we cannot conclude that the relationships we have identified are causal, and therefore they need confirmation. Finally, we conducted many statistical tests, creating a risk for type one errors.

It is important to note that it is not known whether the relationships observed in this study, are transient or if they are maintained after early infancy. A study in children of Indian mothers found that at two and five [27] as well as at 9.5 years of age [28], girls with mothers with diabetes had larger skinfolds than girls with healthy mothers, while no such difference was found for boys. This suggests that the sex difference we have observed also persists during childhood. However, the study by Krishnevi *et al.* [27,28] is small, and extended studies in this area are needed.

Using accurate methodology this study confirmed previous results [3], showing that the blood glucose concentration of pregnant women is related to the weight and fatness of their babies. Furthermore, based on accurate body composition methodology, our results suggest that glucose homeostasis variables in the circulation of pregnant women are related to their own body composition as well as to that of their infants. Finally, our data indicate that the increased insulin resistance associated with maternal fatness is a factor mediating a previously identified relationship between fatness of mothers and fatness of daughters.

## References

[1] World Health Organization Childhood Overweight and Obesity. http://www.who.int/dietphysicalactivity/childhood/en/.

[2] Yu Z.B., Han S.P., Zhu G.Z., Zhu C., Wang X.J., Cao X.G., Guo X.R. (2011). Birth weight and subsequent risk of obesity: A systematic review and meta-analysis. Obes. Rev..

[3] HAPO Study Cooperative Research Group (2009). Hyperglycemia and Adverse Pregnancy Outcome (HAPO) Study: Associations with neonatal anthropometrics. Diabetes.

[4] Henriksson P., Löf M., Forsum E. (2015). Parental fat-free mass is related to fat-free mass of infants and maternal fat mass is related to fat mass of infant girls. Acta Paediatr..

[5] Lingwood B.E., Henry A.M., d’Emden M.C., Fullerton A.M., Mortimer R.H., Colditz P.B., Lê Cao K.A., Callaway L.K. (2011). Determinants of body fat in infants of women with gestational diabetes mellitus differ with fetal sex. Diabetes Care.

[6] Friis C.M., Qvigstad E., Paasche Roland M.C., Godang K., Voldner N., Bollerslev J., Henriksen T. (2013). Newborn body fat: Associations with maternal metabolic state and placental size. PLoS ONE.

[7] Olausson H., Löf M., Brismar K., Forsum E., Sohlström A. (2010). Maternal serum concentrations of insulin-like growth factor (IGF)-I and IGF binding protein-1 before and during pregnancy in relation to maternal body weight and composition and infant birth weight. Br. J. Nutr..

[8] Olausson H., Löf M., Brismar K., Lewitt M., Forsum E., Sohlström A. (2008). Longitudinal study of the maternal insulin-like growth factor system before, during and after pregnancy in relation to fetal and infant weight. Horm. Res..

[9] Åsvold B.O., Eskild A., Jenum P.A., Vatten L.J. (2011). Maternal concentrations of insulin-like growth factor I and insulin-like growth factor binding protein 1 during pregnancy and birth weight of offspring. Am. J. Epidemiol..

[10] Murphy L.J. (2003). The role of the insulin-like growth factors and their binding proteins in glucose homeostasis. Exp. Diabetes Res..

[11] Lindsay C.A., Huston L., Amini S.B., Catalano P.M. (1997). Longitudinal changes in the relationship between body mass index and percent body fat in pregnancy. Obstet. Gynecol..

[12] Knight B., Shields B.M., Hill A., Powell R.J., Wright D., Hattersley A.T. (2007). The impact of maternal glycemia and obesity on early postnatal growth in a nondiabetic Caucasian population. Diabetes Care.

[13] Jörgensen C., Sjöberg S.O. (1997). Fetometri och graviditetsbestämning. Obstetriska Ultraljud.

[14] Henriksson P., Löf M., Forsum E. (2013). Assessment and prediction of thoracic gas volume in pregnant women: An evaluation in relation to body composition assessment using air displacement plethysmography. Br. J. Nutr..

[15] Van Raaij J.M., Peek M.E., Vermaat-Miedema S.H., Schonk C.M., Hautvast J.G. (1988). New equations for estimating body fat mass in pregnancy from body density or total body water. Am. J. Clin. Nutr..

[16] Forsum E., Henriksson P., Löf M. (2014). The two-component model for calculating total body fat from body density: An evaluation in healthy women before, during and after pregnancy. Nutrients.

[17] Jeppsson J.O., Kobold U., Barr J., Finke A., Hoelzel W., Hoshino T., Miedema K., Mosca A., Mauri P., Paroni R. (2002). Approved IFCC reference method for the measurement of HbA1c in human blood. Clin. Chem. Lab. Med..

[18] Matthews D.R., Hosker J.P., Rudenski A.S., Naylor B.A., Treacher D.F., Turner R.C. (1985). Homeostasis model assessment: Insulin resistance and β-cell function from fasting plasma glucose and insulin concentrations in man. Diabetologia.

[19] Ellis K.J., Yao M., Shypailo R.J., Urlando A., Wong W.W., Heird W.C. (2007). Body-composition assessment in infancy: Air-displacement plethysmography compared with a reference 4-compartment model. Am. J. Clin. Nutr..

[20] Kleinbaum D.G., Kupper L.L., Nizam A., Muller K.E. (2007). Applied Regression Analysis and Other Multivariable Methods.

[21] International Association of Diabetes and Pregnancy Study Groups Consensus Panel (2010). International association of diabetes and pregnancy study groups recommendations on the diagnosis and classification of hyperglycemia in pregnancy. Diabetes Care.

[22] Eriksson B., Löf M., Olausson H., Forsum E. (2010). Body fat, insulin resistance, energy expenditure and serum concentrations of leptin, adiponectin and resistin before, during and after pregnancy in healthy Swedish women. Br. J. Nutr..

[23] Watve M.G., Yajnik C.S. (2007). Evolutionary origins of insulin resistance: A behavioural switch hypothesis. BMC Evol. Biol..

[24] Roland M.C., Friis C.M., Godang K., Bollerslev J., Haugen G., Henriksen T. (2014). Maternal factors associated with fetal growth and birthweight are independent determinants of placental weight and exhibit differential effects by fetal sex. PLoS ONE.

[25] Brett K.E., Ferraro Z.M., Yockell-Lelievre J., Gruslin A., Adamo K.B. (2014). Maternal-fetal nutrient transport in pregnancy pathologies: The role of the placenta. Int. J. Mol. Sci..

[26] Simon-Muela I., Naf S., Ballesteros M., Vendrell J., Ceperuelo-Mallafre V., de la Flor M., Megia A. (2013). Gender determines the actions of adiponectin multimers on fetal growth and adiposity. Am. J. Obstet. Gynecol..

[27] Krishnaveni G.V., Hill J.C., Leary S.D., Veena S.R., Saperia J., Saroja A., Karat S.C., Fall C.H. (2005). Anthropometry, glucose tolerance, and insulin concentrations in Indian children: Relationships to maternal glucose and insulin concentrations during pregnancy. Diabetes Care.

[28] Krishnaveni G.V., Veena S.R., Hill J.C., Kehoe S., Karat S.C., Fall C.H. (2010). Intrauterine exposure to maternal diabetes is associated with higher adiposity and insulin resistance and clustering of cardiovascular risk markers in Indian children. Diabetes Care.

